# Steroid-refractory immune mediated hepatitis managed with budesonide in patients with metastatic melanoma: proof of concept and literature review

**DOI:** 10.1093/oncolo/oyae361

**Published:** 2025-01-20

**Authors:** Roma A Kankaria, Douglas B Johnson

**Affiliations:** Division of Hematology/Oncology, Department of Medicine, Vanderbilt University School of Medicine, Nashville, TN 37232, United States; Division of Hematology/Oncology, Department of Medicine, Vanderbilt University Medical Center, Nashville, TN 37232, United States

**Keywords:** metastatic melanoma, immunotherapy, toxicity treatment, refractory hepatitis, budesonide

## Abstract

Immune checkpoint inhibitors (ICIs) have advanced the treatment of metastatic melanoma. However, some patients develop ICI-associated toxicities like hepatitis (ie, immune-mediated hepatitis; IMH). Although these toxicities usually resolve with steroids, steroid-refractory events may occur, which may be a major source of morbidity and mortality without obviously defined treatment algorithms. Herein, we present 2 patients with metastatic melanoma who had IMH that was steroid-refractory and only partially mycophenolate-responsive, but fully resolved with budesonide. The case suggests that budesonide is a potential option to treat IMH that is refractory to standard treatments, but further investigation in a larger series is needed to identify the most optimal setting for budesonide use.

## Introduction

Immune checkpoint inhibitors (ICIs) are monoclonal antibodies that target programmed cell death-1 (PD-1), cytotoxic T-lymphocyte-associated protein-4 (CTLA-4), and other immune system down-regulators. They have demonstrated durable responses in patients with advanced melanoma, with a 5-year survival of 35%-50%.^1^ However, ICIs cause immune-related adverse events (irAEs), autoinflammatory toxicities that can impact many organ systems.

Immune-mediated hepatitis (IMH) is one such inflammatory condition that may complicate therapy. High-grade IMH occurs in approximately 5%-10% of patients treated with a combination of ipilimumab and nivolumab, and 1%-2% of patients treated with anti-programmed death-1 (PD-1) monotherapy.^1^ Although steroids often reverse this inflammation, mycophenolate mofetil or other immunosuppressants may be required for steroid-refractory cases.^2-5^ The most effective treatment for mycophenolate-refractory cases remains unclear; further, immunosuppressant treatment could be associated with infectious risks and potential concerns for disease progressions. Here, we describe 2 patients with IMH refractory to high-dose corticosteroids and mycophenolate who responded to budesonide, a therapy with a potentially attractive safety and efficacy profile for IMH. After reviewing these cases, we place them in the context of the larger field of IMH and other forms of immune-mediated hepatitis.

## Case description

### Case 1

A 50-year-old female presented with a localized *BRAF*^V600E^-mutated melanoma (non-ulcerated, Breslow thickness 2 mm) that was treated with wide local excision and sentinel lymph node (SLN) biopsy. Eleven months later, imaging demonstrated bulky iliac adenopathy, biopsy-confirmed as metastatic melanoma. She received a combination ipilimumab and nivolumab and experienced a partial response after 4 cycles, with nearly resolved inguinal adenopathy. However, she developed biochemical evidence of IMH, with elevated aspartate aminotransferase or AST of 165 U/L (normal 0-39 U/L) and alanine transaminase or ALT of 371 U/L (normal 0-40 U/L); bilirubin (0.3mg/dl) and alkaline phosphatase were normal (74 units/L; normal 40-150 units/L) and remained normal, liver biopsy was not done.

**Figure 1.**
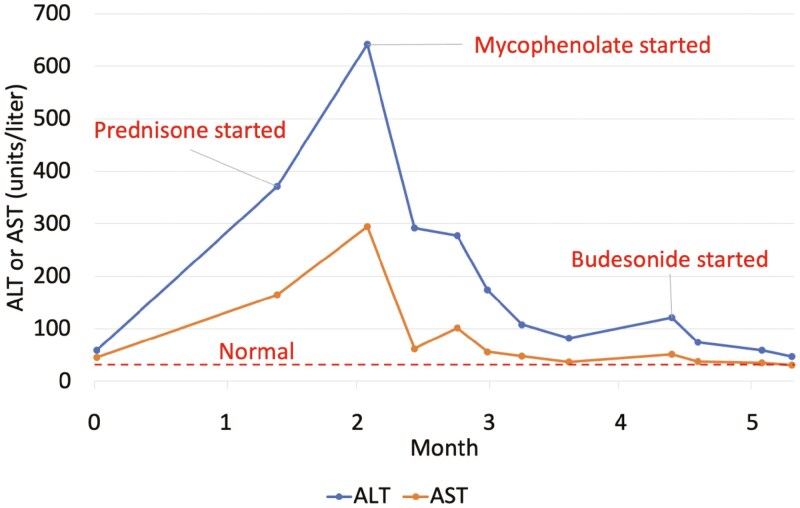
Changes in liver enzymes and medication administration over time for patient 1 with ICI-induced hepatitis.

She received prednisone 1 mg/kg, but liver enzymes increased (AST 294 U/L and ALT 642 U/L), and she was treated with mycophenolate mofetil 1 gram twice daily with initial improvement and near normalization of liver enzymes (**Figure 1**). Following steroid taper (while still on mycophenolate), her liver enzymes increased again (AST 51 U/L and ALT 121 U/L), and she was started on budesonide 9 mg daily. Her liver enzymes normalized over the next 3 weeks and her mycophenolate was discontinued, followed by budesonide taper (6 mg for 1 week, then 3 mg for 1 week) with continued resolution off all therapy now 6 months later.

### Case 2

A 66-year-old male presented with an *NRAS*-mutated melanoma (ulcerated, Breslow thickness 4 mm) on the lower mid-back that was treated with wide local excision and SLN biopsy (0/3 involved lymph nodes). PET and MRI of the head were negative for distant metastasis. Fifteen months later, he developed a new subcutaneous lesion on the flank that was found to be recurrent in-transit metastasis of the melanoma, which was resected. He completed 1 year of adjuvant pembrolizumab without toxicities. One and a half years after finishing pembrolizumab, he developed bilateral adrenal nodules suspicious for metastatic disease on CT; a biopsy confirmed metastatic melanoma.

**Figure 2.**
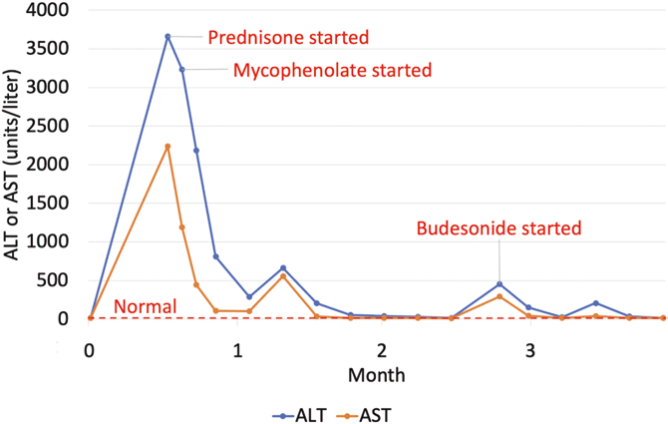
Changes in liver enzymes and medication administration over time for patient 2 with ICI-induced hepatitis.

The patient began a combination of ipilimumab and nivolumab. After 3 doses, he developed fevers and was found to have high-grade IMH, with AST 2238 U/L, ALT 3662 U/L, alkaline phosphatase (alk phos) 198 (normal range 30-120), and normal bilirubin (Figure 2). The acute viral hepatitis panel was negative; a liver biopsy was not done. The patient’s liver enzymes had only minimal improvement with prednisone 1 mg/kg, so mycophenolate mofetil 1 gram twice daily was added; his liver enzymes normalized. However, after a 5-week prednisone taper, his liver enzymes increased again (AST 294 U/L and ALT 453 U/L). He was started on budesonide 9 mg daily, and his liver enzymes normalized over the subsequent weeks, followed by mycophenolate then budesonide taper over about 4 weeks. He has continued resolution 5 months after all therapy was discontinued and has an ongoing partial response for his melanoma.

## Discussion and review

Patients treated with combined anti-CTLA-4 and anti-PD-1 have rates of any-grade IMH of 19%-37% and rates of grade 3+ IMH of 9%-16%. Our report highlights 2 patients with IMH who were steroid-refractory and only partially mycophenolate-responsive, but fully resolved with budesonide, allowing for immunosuppressive discontinuation. The study suggests that budesonide could potentially be an effective agent for IMH, although more data are needed before its more widespread use.

Budesonide is a potentially promising treatment for ICI-associated hepatitis because of its metabolism and side effect profile. This agent has become a standard of care agent for autoimmune hepatitis (often in combination with other agents like azathioprine).^6^ Enteric-coated budesonide is not degraded by gastric acid, which allows the drug to reach the ileum and be absorbed. It then undergoes hepatic first-pass metabolism to compounds that have minimal to no steroid activity (6β-hydroxybudesonide and 16α-hydroxyprednisolone), thus limiting its effects on the intestine and liver. As a result of its minimal systemic absorption, the drug also causes fewer systemic side effects than other glucocorticoids (primarily studied in the context of inflammatory bowel disease or autoimmune hepatitis) and does not require pneumocystis prophylaxis or entail significant risk of infection.^7,8^ In the context of ICI therapy, limiting systemic steroid exposure could be important, as some studies have suggested that high doses of systemic steroids, as well as early onset, may compromise response to ICI treatment.^9-12^

Recent case reports have also shown IMH effectively managed with budesonide,^13,14^ although one series employed budesonide as a prophylactic during ICI re-treatment. However, our series are the first cases to the best of our knowledge in patients with steroid-refractory, mycophenolate (partially) non-responsive hepatitis (or perhaps poorly responsive in the first case). Interestingly, a recent study corroborated the potential efficacy of budesonide in other steroid-refractory settings; 19 patients with immune-related enteritis (approximately half with concurrent colitis) were treated with open capsule budesonide (to facilitate proximal intestinal absorption), with 18 patients experiencing clinical improvement.^15^ Concurrently, these studies suggest that These data are particularly important in that the best treatments for steroid and/or mycophenolate-refractory (or poorly responsive) IMH are not well-defined. Further, predicting which patients will have steroid-refractory IMH is not well-defined either, although patients with cholestatic patterns may be at higher risk (which does not include either patient in this series).^16-18^ It should be noted that biopsies were not done in either case, thus adding a small amount of diagnostic uncertainty. However, the timing of combination immune therapy and lack of other risk factors suggest an extremely high likelihood of IMH.

These data also bring up the possibility that budesonide could preclude the use of high-dose prednisone or other systemic steroids. In addition to the data already discussed, several other pieces of evidence could support this. First, topical treatments are well-described and highly effective for cutaneous toxicities from ICI therapy.^19^ Second, modest-sized case series have reported relatively high remission rates from budesonide alone of immune-related enteritis/colitis (>50% remission rates).^15,20^ Finally, the experience both with autoimmune hepatitis and our limited series with IMH provides still additional evidence. Ideally, prospective clinical trials should be done to define the most appropriate therapies. In the absence of such high-quality data, careful treatment and close follow-up could perhaps be cautiously considered on a case-by-case basis. More severe cases, including when synthetic function is compromised, should not be treated with budesonide.

Our cases and similar reports demonstrate that budesonide may be a possible option to treat ICI-associated hepatitis to spare systemic steroid effects. However, more research is needed to identify the most optimal setting (whether used in lieu of steroids or in refractory cases). More data is also needed on the efficacy of co-administration of budesonide when challenging with ICI in patients with hepatitis.

## Data Availability

A data availability statement is not applicable for this case series.
